# Chinese expert consensus on the diagnosis and treatment of thymic epithelial tumors

**DOI:** 10.1111/1759-7714.14847

**Published:** 2023-03-16

**Authors:** Chunwei Xu, Yongchang Zhang, Wenxian Wang, Qian Wang, Ziming Li, Zhengbo Song, Jiandong Wang, Jinpu Yu, Jingjing Liu, Shirong Zhang, Xiuyu Cai, Ming Wu, Ping Zhan, Hongbing Liu, Tangfeng Lv, Liyun Miao, Lingfeng Min, Jiancheng Li, Baogang Liu, Jingping Yuan, Zhansheng Jiang, Gen Lin, Xiaohui Chen, Xingxiang Pu, Chuangzhou Rao, Dongqing Lv, Zongyang Yu, Xiaoyan Li, Chuanhao Tang, Chengzhi Zhou, Junping Zhang, Hui Guo, Qian Chu, Rui Meng, Xuewen Liu, Jingxun Wu, Xiao Hu, Min Fang, Jin Zhou, Zhengfei Zhu, Xiaofeng Chen, Weiwei Pan, Fei Pang, Yuxiang Zhou, Qijie Jian, Kai Wang, Liping Wang, Youcai Zhu, Guocai Yang, Xinqing Lin, Jing Cai, Lijun Liang, Huijing Feng, Lin Wang, Yingying Du, Wang Yao, Xuefei Shi, Xiaomin Niu, Dongmei Yuan, Yanwen Yao, Jianhui Huang, Yinbin Zhang, Pingli Sun, Hong Wang, Mingxiang Ye, Dong Wang, Zhaofeng Wang, Yue Hao, Zhen Wang, Bing Wan, Donglai Lv, Genhua Yu, Anna Li, Jin Kang, Jiatao Zhang, Chao Zhang, Huafei Chen, Lin Shi, Leiguang Ye, Gaoming Wang, Yina Wang, Feng Gao, Wei Zhou, Chunxiu Hu, Jianguo Wei, Bihui Li, Zhongwu Li, Yuan Li, Zhefeng Liu, Nong Yang, Lin Wu, Qiming Wang, Wenbin Huang, Zhuan Hong, Guansong Wang, Meiyu Fang, Yong Fang, Xixu Zhu, Kaiqi Du, Jiansong Ji, Yi Shen, Yiping Zhang, Shenglin Ma, Yong Song, Yuanzhi Lu, Anwen Liu, Wenfeng Fang, Wenzhao Zhong

**Affiliations:** ^1^ Institute of Cancer and Basic Medicine (ICBM) Chinese Academy of Sciences Hangzhou People's Republic of China; ^2^ Department of Chemotherapy Chinese Academy of Sciences University Cancer Hospital (Zhejiang Cancer Hospital) Zhejiang People's Republic of China; ^3^ Department of Respiratory Medicine, Affiliated Jinling Hospital Medical School of Nanjing University Nanjing People's Republic of China; ^4^ Department of Medical Oncology Lung Cancer and Gastrointestinal Unit, Hunan Cancer Hospital/The Affiliated Cancer Hospital of Xiangya School of Medicine, Central South University Changsha People's Republic of China; ^5^ Department of Respiratory Medicine Affiliated Hospital of Nanjing University of Chinese Medicine, Jiangsu Province Hospital of Chinese Medicine Nanjing People's Republic of China; ^6^ Department of Shanghai Lung Cancer Center, Shanghai Chest Hospital Shanghai Jiao Tong University Shanghai People's Republic of China; ^7^ Department of Pathology, Affiliated Jinling Hospital Medical School of Nanjing University Nanjing People's Republic of China; ^8^ Department of Cancer Molecular Diagnostics Core Tianjin Medical University Cancer Institute and Hospital Tianjin People's Republic of China; ^9^ Department of Thoracic Cancer Jilin Cancer Hospital Jilin People's Republic of China; ^10^ Translational Medicine Research Center, Key Laboratory of Clinical Cancer Pharmacology and Toxicology Research of Zhejiang Province, Affiliated Hangzhou First People's Hospital, Cancer Center Zhejiang University School of Medicine Hangzhou People's Republic of China; ^11^ Department of VIP Inpatient, Sun Yet‐Sen University Cancer Center, State Key Laboratory of Oncology in South China Collaborative Innovation Center for Cancer Medicine Guangzhou People's Republic of China; ^12^ Department of Thoracic Surgery, Second Affiliated Hospital of Zhejiang University School of Medicine, Cancer Center Zhejiang University Hangzhou People's Republic of China; ^13^ Department of Respiratory Medicine, Affiliated Drum Tower Hospital Medical School of Nanjing University Nanjing People's Republic of China; ^14^ Department of Respiratory Medicine Clinical Medical School of Yangzhou University, Subei People's Hospital of Jiangsu Province Yangzhou People's Republic of China; ^15^ Department of Radiation Oncology Fujian Medical University Cancer Hospital & Fujian Cancer Hospital Fuzhou People's Republic of China; ^16^ Department of Oncology Harbin Medical University Cancer Hospital Harbin People's Republic of China; ^17^ Department of Pathology Renmin Hospital of Wuhan University Wuhan People's Republic of China; ^18^ Derpartment of Integrative Oncology Tianjin Medical University Cancer Institute and Hospital Tianjin People's Republic of China; ^19^ Department of Medical Oncology Fujian Medical University Cancer Hospital & Fujian Cancer Hospital Fuzhou People's Republic of China; ^20^ Department of Thoracic Surgery Fujian Medical University Cancer Hospital & Fujian Cancer Hospital Fuzhou People's Republic of China; ^21^ Department of Medical Oncology, Hunan Cancer Hospital/The Affiliated Cancer Hospital of Xiangya School of Medicine Central South University Changsha People's Republic of China; ^22^ Department of Radiotherapy and Chemotherapy, Hwamei Hospital University of Chinese Academy of Sciences Ningbo People's Republic of China; ^23^ Department of Pulmonary Medicine Taizhou Hospital of Wenzhou Medical University Taizhou People's Republic of China; ^24^ Department of Respiratory Medicine, the 900th Hospital of the Joint Logistics Team (the Former Fuzhou General Hospital) Fujian Medical University Fuzhou People's Republic of China; ^25^ Department of Oncology Beijing Tiantan Hospital, Capital Medical University Beijing People's Republic of China; ^26^ Department of Medical Oncology Peking University International Hospital Beijing People's Republic of China; ^27^ State Key Laboratory of Respiratory Disease, National Clinical Research Center for Respiratory Disease, Guangzhou Institute of Respiratory Health The First Affiliated Hospital of Guangzhou Medical University(The First Affiliated Hospital of Guangzhou Medical University) Guangzhou People's Republic of China; ^28^ Department of Thoracic Oncology Shanxi Academy of Medical Sciences, Shanxi Bethune Hospital Taiyuan People's Republic of China; ^29^ Department of Medical Oncology The First Affiliated Hospital of Xi'an Jiaotong University Xi'an People's Republic of China; ^30^ Department of Oncology, Tongji Hospital of Tongji Medical College Huazhong University of Science and Technology Wuhan People's Republic of China; ^31^ Cancer Center, Union Hospital, Tongji Medical College Huazhong University of Science and Technology Wuhan People's Republic of China; ^32^ Department of Oncology, the Third Xiangya Hospital Central South University Changsha People's Republic of China; ^33^ Department of Medical Oncology, the First Affiliated Hospital of Medicine Xiamen University Xiamen People's Republic of China; ^34^ Zhejiang Key Laboratory of Radiation Oncology Cancer Hospital of the University of Chinese Academy of Sciences (Zhejiang Cancer Hospital) Zhejiang People's Republic of China; ^35^ Department of Medical Oncology, Sichuan Cancer Hospital & Institute, Sichuan Cancer Center, School of Medicine University of Electronic Science and Technology Chengdu People's Republic of China; ^36^ Department of Radiation Oncology Fudan University Shanghai Cancer Center Shanghai People's Republic of China; ^37^ Department of Oncology Jiangsu Province Hospital and Nanjing Medical University First Affiliated Hospital Nanjing People's Republic of China; ^38^ Department of Cell Biology, College of Medicine Jiaxing University Jiaxing People's Republic of China; ^39^ Department of Medical Shanghai OrigiMed Co, Ltd Shanghai People's Republic of China; ^40^ Department of Oncology Baotou Cancer Hospital Baotou People's Republic of China; ^41^ Department of Thoracic Disease Diagnosis and Treatment Center, Zhejiang Rongjun Hospital The Third Affiliated Hospital of Jiaxing University Jiaxing People's Republic of China; ^42^ Department of Thoracic Surgery Zhoushan Hospital, Wenzhou Medical University Zhejiang People's Republic of China; ^43^ Department of Oncology Second Affiliated Hospital of Nanchang University Nanchang People's Republic of China; ^44^ Department of Pathology, Shanxi Academy of Medical Sciences Shanxi Bethune Hospital Taiyuan People's Republic of China; ^45^ Department of Oncology The First Affiliated Hospital of Anhui Medical University Hefei People's Republic of China; ^46^ Department of Interventional Oncology, The First Affiliated Hospital Sun Yat‐sen University Guangzhou People's Republic of China; ^47^ Department of Respiratory Medicine, Huzhou Hospital Zhejiang University School of Medicine Huzhou People's Republic of China; ^48^ Department of Oncology Lishui Municipal Central Hospital Lishui People's Republic of China; ^49^ Department of Oncology the Second Affiliated Hospital of Medical College, Xi'an Jiaotong University Xi'an People's Republic of China; ^50^ Department of Pathology The Second Hospital of Jilin University Changchun People's Republic of China; ^51^ Senior Department of Oncology The 5th Medical Center of PLA General Hospital Beijing People's Republic of China; ^52^ Department of Radiation Oncology, Affiliated Jinling Hospital Medical School of Nanjing University Nanjing People's Republic of China; ^53^ Department of Respiratory Medicine The Affiliated Jiangning Hospital of Nanjing Medical University Nanjing People's Republic of China; ^54^ Department of Clinical Oncology The 901 Hospital of Joint Logistics Support Force of People Liberation Army Hefei People's Republic of China; ^55^ Department of Radiation Oncology Zhebei Mingzhou Hospital Huzhou People's Republic of China; ^56^ Guangdong Lung Cancer Institute Guangdong Provincial Laboratory of Translational Medicine in Lung Cancer, Guangdong Provincial People's Hospital, Guangdong Academy of Medical Sciences, School of Medicine Guangzhou People's Republic of China; ^57^ Department of Respiratory Medicine Zhongshan Hospital, Fudan University Shanghai People's Republic of China; ^58^ Department of Thoracic Surgery Xuzhou Central Hospital, Xuzhou Clinical School of Xuzhou Medical University Xuzhou People's Republic of China; ^59^ Department of Oncology The First Affiliated Hospital, College of Medicine, Zhejiang University Hangzhou People's Republic of China; ^60^ Department of Thoracic Surgery The Fourth Hospital of Hebei Medical University Shijiazhuang People's Republic of China; ^61^ Department of Cancer Radiotherapy and Chemotherapy Zhejiang Queue Hospital Quzhou People's Republic of China; ^62^ Department of Pahtology Shaoxing People's Hospital (Shaoxing Hospital, Zhejiang University School of Medicine) Shaoxing People's Republic of China; ^63^ Department of Oncology The Second Affiliated Hospital of Guilin Medical University Guilin People's Republic of China; ^64^ Key Laboratory of Carcinogenesis and Translational Research (Ministry of Education/Beijing), Department of Pathology Peking University Cancer Hospital & Institute Beijing People's Republic of China; ^65^ Department of Pathology Fudan University Shanghai Cancer Center Shanghai People's Republic of China; ^66^ Department of Internal Medicine The Affiliated Cancer Hospital of Zhengzhou University Henan Cancer Hospital Zhengzhou People's Republic of China; ^67^ Department of Pathology the First Affiliated Hospital of Henan University of Science and Technology Luoyang People's Republic of China; ^68^ Department of Medical Oncology, Jiangsu Cancer Hospital Nanjing Medical University Affiliated Cancer Hospital Nanjing People's Republic of China; ^69^ Institute of Respiratory Diseases Xinqiao Hospital, Third Military Medical University Chongqing People's Republic of China; ^70^ Department of Medical Oncology Sir Run Run Shaw Hospital, Zhejiang University Hangzhou People's Republic of China; ^71^ Department of Radiology Lishui Municipal Central Hospital Lishui People's Republic of China; ^72^ Department of Thoracic Surgery, Affiliated Jinling Hospital Medical School of Nanjing University Nanjing People's Republic of China; ^73^ Department of Oncology Key Laboratory of Clinical Cancer Pharmacology and Toxicology Research of Zhejiang Province, Affiliated Hangzhou Cancer Hospital, Cancer Center, Zhejiang University School of Medicine Hangzhou People's Republic of China; ^74^ Department of Clinical Pathology The First Affiliated Hospital Of Jinan University Guangzhou People's Republic of China; ^75^ Department of Medical Oncology Sun Yat‐sen University Cancer Center, State Key Laboratory of Oncology in South China, Collaborative Innovation Center for Cancer Medicine Guangzhou People's Republic of China

**Keywords:** diagnosis, thymic carcinoma, thymic epithelial tumor, thymoma, treatment

## Abstract

Thymic epithelial tumors (TETs) are a relatively rare type of thoracic tumor, accounting for less than 1% of all tumors. The incidence of TETs is about 3.93/10000 in China, slightly higher than that of European and American countries. For resectable TETs, complete surgical resection is recommended. Radiotherapy or chemotherapy may be used as postoperative adjuvant treatment. Treatment for advanced, unresectable TETs consist mainly of radiotherapy and chemotherapy, but there is a lack of standard first‐ and second‐line treatment regimens. Recently, targeted therapies and immune checkpoint inhibitors have shown promising outcomes in TETs. Based on the currently available clinical evidences and the opinions of the national experts, the Thymic Oncology Group of Yangtze River Delta Lung Cancer Cooperation Group (East China LUng caNcer Group, ECLUNG; Youth Committee) established this Chinese expert consensus on the clinical diagnosis and treatment of TETs, covering the epidemiology, diagnosis, treatment, prognosis and follow‐up of TETs.

## INTRODUCTION

Thymic epithelial tumors (TETs) are a relatively rare type of thoracic solid tumor. The incidence of TETs is about 3.93/10 000 in China. At present, all TET subtypes are considered to have malignant potential. Even patients with type A thymoma (TM) may develop distant metastasis, and completely resected early TETs may still recur. On the other hand, TET is a relatively inert tumor. Patients may still survive for quite a long time after disease progression or recurrence, making it extremely difficult to carry out large‐scale prospective randomized studies to obtain high‐quality evidence and guide clinical practice. Therefore, the diagnosis and treatment of TETs remain controversial, with empirical diagnosis and treatment as the mainstay. The current National Comprehensive Cancer Network (NCCN) guidelines are also mainly based on expert opinions. In recent years, global and regional clinical studies have led to encouraging results, in which China's studies also play an important role.

Currently, there are already many guidelines and consensus available, providing guidance for the standard diagnosis and treatment of TETs. However, there are still many issues to be addressed in clinical practice, especially in the standardization of the application of new diagnostic techniques and therapies. To promote the development of diagnosis and treatment and improve clinical practice of TETs, the Thymic Oncology Group of Yangtze River Delta Lung Cancer Cooperation Group (East China LUng caNcer Group, ECLUNG; Youth Committee) organized a discussion panel comprising relevant experts in the field of thymic tumor in China and carried out in‐depth discussions based on existing literature evidence and NCCN/European Society of Medical Oncology (ESMO) guidelines. We finally reached a consensus on the diagnosis and treatment of TETs, aiming to turn recent developments to the benefit of patients in clinical practice.

## INCIDENCE, ETIOLOGY AND SCREENING

Consensus 1: The incidence of TET is low, and low‐dose computed tomography (CT) is currently not recommended for screening of TET (Recommended).

### Incidence

Thymic epithelial tumors (TETs) are a rare solid tumor with an incidence of 1.3–3.2/10 000, and are a common type of anterior mediastinal tumor. According to the degree of malignancy, TETs can mainly be divided into two types: TM and thymic carcinoma (TC). The China Cancer Registry Annual Report released in 2019 reported that there were 1562 new cases of malignant thymic tumors (ICD10 code: C37), and the incidence and standardized incidence rates (Segi's world standard population) were 4.09/10 000 and 2.73/10 000, respectively. The incidence in China is higher than that in European and American countries, but is equivalent to other Asian countries. A retrospective study from Japan reviewing the thymic cancer patients in the national database from 2009 to 2015 revealed that the incidence rate of TC was about 2.9/10 000.^1^ In a 19‐year‐long study from South Korea, 5812 cases of TET were diagnosed, and the incidence rates of TM and TC were 3/10 000 and 5/10 000, respectively.^2^ The incidence rate of thymic tumors in North America (2.14/10 000) is lower than that in Asian populations.^3^ Among them, there are differences in the incidence rate of Caucasians (1.89/10 000) and Asians (3.74/10 000). The above data indicate that the incidence rate of TET is race‐specific. In the past 20 years, the incidence rate of TET had an increasing trend in China. This trend is partly due to the advances of diagnostic technologies, the improvement of instrument sensitivity and the increase in understanding of TET.

### Etiology and screening

The etiology of TET remains unclear. Drinking, smoking and ionizing radiation are not risk factors of TETs. The high incidence of TM among African Americans and Asians and Pacific islanders suggests that TETs may be hereditary.

At present, there is no data supporting that the formation of TETs can be prevented. There is also no evidence indicating that low‐dose CT screening can improve the prognosis of patients with TM and TC. Considering the low incidence of TET, low‐dose CT is not recommended for routine screening of TET. However, for patients diagnosed with autoimmune diseases such as myasthenia gravis (MG), screening of TET can be performed through enhanced chest CT.

For small anterior mediastinal nodules (generally defined as ≤3 cm in diameter) found by body check or accident, no suggestion for management is given in both the NCCN and ESMO guidelines, and the differential diagnosis requires the combination of chest CT and magnetic resonance imaging (MRI). For suspected benign occupying lesions (thymic cyst, thymic hyperplasia/involution, small lymph nodes, etc.), it is recommended that re‐examination by CT or MRI is performed after 3–6 months, and once every 1–2 years thereafter to avoid unnecessary surgery; for suspected TETs with high‐risk histological subtypes (B2/B3 TM, TC), direct surgery is recommended; for suspected low‐risk TM (A/AB/B1), surgery or active surveillance is recommended because the edge of such small nodules is clear without external invasion and the tumor doubling time can be longer than 1 year, meaning that it is theoretically safe to re‐examine 6 months after the first diagnosis.

According to previous studies, many early thymic tumors suspected to be benign may also have the possibility of recurrence and metastasis after surgery. Therefore, all thymic tumors are now considered as malignant. In March 2015, the International Agency for Research on Cancer released the World Health Organization (WHO) classification of TET. The classification is based on the reached consensus regarding thymic tumor at the multidisciplinary seminar organized by the International Thymic Malignancy Interest Group (ITMIG) in December 2011. Thymic tumors are inert tumors. Even after disease progression, some TM patients still have a relatively long survival time, with a 5‐year overall survival (OS) of almost 90%. Therefore carrying out long‐term follow‐up (e.g., 10 years) for TETs is recommended in order to better study the OS and recurrence of patients. In contrast, TC is often accompanied by distant metastasis, and the 5‐year OS is only 55%.

## DIAGNOSIS

Consensus 2: Enhanced chest CT (mediastinal window) is the preferred imaging method for diagnosis of thymic tumors. MRI, positron emission tomography/CT (PET/CT) and other imaging methods have unique characteristics and advantages, and can be used if conditions permit (strongly recommended).

Consensus 3: CT‐guided needle biopsy is recommended as a standard operation, and ultrasound‐guided needle biopsy or thoracoscopic examination is recommended as a supplementary operation under specific circumstances (strongly recommended).

Consensus 4: Histopathological examination is the gold standard for the diagnosis of TETs. Because histological subtypes are associated with prognosis, histological subtypes should be determined by pathological examination of tissue samples. Immunohistochemistry and next‐generation sequencing are also valuable for differentiating TET subtypes (strongly recommended).

### Clinical diagnosis

The diagnosis should be based on a comprehensive analysis of the patient's medical history and clinical manifestations. The onset of thymic tumor is mostly occult. When the tumor volume is small, the patient may have no symptoms; for larger tumors, local chest symptoms caused by tumor invasion or compression of adjacent mediastinal structures are the major symptoms, including cough, chest pain, chest tightness, shortness of breath, head and face swelling, etc. Some TM patients have concomitant autoimmune diseases. The most common comorbidity is MG. MG is common in type AB, B1 and B2 TM, and is often related to antiacetylcholine receptor antibody. Other common concomitant diseases include pure red aplastic anemia and low γ globulinemia. TM in patients is suspected when autoimmune disease is accompanied by an anterior mediastinal mass. For TC patients, nonspecific local stimulation or compression symptoms are common. When a tumor invades the lung and bronchus, patients may have severe cough, dyspnea and other symptoms; when it compresses the sympathetic nerve, it can cause ipsilateral blepharoptosis, pupillary narrowing, enophthalmos, forehead sweatlessness and Horner syndrome and when it compresses the recurrent laryngeal nerve, it can cause hoarseness. When the superior vena cava is compressed, it can cause superior vena cava obstruction syndrome.

### Imaging diagnosis

Enhanced chest CT scan (mediastinal window) is the first choice for imaging diagnosis of thymic tumor. It is capable of examining the extent of tumor lesions, discovering peripheral tissue infiltration and distant metastasis and predicting the tumor stage, and thus plays a critical role in guiding the treatment decision and predicting the prognosis of TM. From the perspective of imaging, TM appears as a tumor with clear boundary in the anterior superior mediastinum. It is capsulated and has uniform density. If the tumor has hemorrhage, necrosis or cyst formation, the imaging manifestation of TM can be pleomorphic. TC often has local infiltration, while regional lymph node metastasis and distant metastasis may also occur. TC is characterized by large anterior mediastinal mass with unclear boundary, often causing exudation and accompanied by pleural effusion and pericardial effusion. Although the imaging features of invasive tumor are vascular injury and unclear demarcation of the surrounding lung tissue, it is difficult to evaluate the invasiveness of thymic tumor by CT. For those patients with unclear CT diagnosis, MRI can be used to evaluate the invasion of tumor into surrounding fat, and accurately distinguish malignant thymic tumor from thymic cyst or thymic hyperplasia, and thus avoiding unnecessary thymectomy. PET/CT has a relatively higher accuracy in early diagnosis and differentiation of benign and malignant TM. Additionally, it can also predict the degree of malignancy to a certain extent. For progressing and advanced tumors, PET‐CT scan can be used to evaluate the distant and systemic metastasis.

### Differential diagnosis

Thymic tumors should be differentiated from other types of tumors in the anterior mediastinum and nonmalignant thymic lesions, including lymphoma, germ cell tumors, physiological thymic hyperplasia, etc. Lymphoma patients usually present with painless lymph node enlargement with or without lactate dehydrogenase elevation; teratoma presents as a mass with uneven density with fat and cystic changes. Seminoma and nonspermatoma are larger. Seminoma is often accompanied by serum β‐human chorionic gonadotropin elevation, and nonspermatoblastoma is often accompanied by alpha‐fetoprotein elevation. It is difficult to distinguish malignant thymic tumor from physiological thymic hyperplasia. Thymic reactive hyperplasia may be caused by stress, injury, chemotherapy, radiotherapy, antihormone or corticosteroid therapy. Thymic lymphohyperplasia is common in patients with MG, and patients with hyperthyroidism, connective tissue disease or vascular disease may also suffer from thymic lymphohyperplasia.

### Pathological diagnosis

WHO classifies TET into type A, AB, B1, B2, B3 and C (TC), which to some extent reflects the biological behavior and prognosis of the tumor.^4^ According to the differences in biological behaviors of tumor subtypes, the histological classification was simplified into three subgroups: low‐risk group (type A, AB and B1), high‐risk group (type B2 and B3) and TC group (type C). Each group possesses different treatment and prognosis. Histopathological classification and radical resection are independent risk factors for prognosis of TET patients **(**Table 1 **)**.

**TABLE 1:** Pathological diagnosis of thymic epithelial tumor (TET).

WHO classification	Diagnostic prerequisites	Other diagnostic criteria
TM		
Type A	A thymic epithelial tumor, with bundle, storiform or pericytoma‐like growth, spindle and oval‐shaped, rarely polygonal‐shaped; Lack of immature TdT‐positive T cells; Most tumors lack necrotic areas, mitotic figures are rare, and Ki‐67 index is low; Atypical type A thymoma can have high proliferation index and focal necrosis	Strong expression of epithelial markers
Type AB	A thymic tumor with lobulated growth pattern; Compose of spindle cell components, Type A lacks lymphocytes and type B is lymphocyte‐rich; Thymic epithelial cells were spindle, oval and focal polygonal‐shaped, with a large amount of immature T cells (focal or diffuse); Spindle cell tumor with moderate TdT‐positive T cell infiltration in more than 10% area	TdT immunohistochemistry assessing TdT‐positive cell density can be used for differential diagnosis from type A thymoma
Type B1	A thymic epithelial tumor with organoid (corticomedullary) structure, mainly composed of cortex; Non‐clustered thymic epithelial cells scattered among a large number of lymphocytes	Scattered epithelial cells with positive cytokeratin, p40 and p63; TdT‐positive immature T cells are distributed in TdT‐negative nodules (medulla) area
Type B2	Lobulated structure; Large amount of lymphocytes; More polygonal or oval‐shaped neoplastic epithelial cells than normal thymic cortex, often appearing in clusters	Scattered or clustered epithelial cells with positive cytokeratin, p40 and p63, and the staining was stronger than that of normal thymic cortex
Type B3	Lobulated structure; Slight or moderate heterotypic and polygonal‐shaped tumor cells are distributed in sheet‐like pattern; Scattered around blood vessels; Lack of lymphocytes	A small amount of immature T cells stained with TdT
Micronodular thymoma with lymphoid stroma	Multiple discrete nodules composed of spindle or oval‐shaped epithelial cells; Abundant epithelial lymphoid stroma	CD20‐positive B cells are dominant in the stroma
Metaplastic thymoma	Biphasic thymoma; Heterotypic unequal, polygonal‐shaped epithelial cells forming interconnected island structures; With spindle cells as the background	
Lipofibroadenoma	A benign thymic tumor similar to breast fibroadenoma, with fat cells and thin epithelial cell bundles, mainly composed of fibrous tissue	
TC (type C)		
Squamous cell carcinoma	An invasive thymic carcinoma, often with fibroproliferative or sclerotic stroma; Exclude adjacent lung cancer invasion or metastasis	Positive CD5, KIT (CD117), FOXN1 and CD205 in immunohistochemistry; *KIT* mutation
Basal cell carcinoma	A basal cell‐like thymic carcinoma with nested shape or cystic spaces, lined with palisade‐shaped basal cell‐like tumor cells	Positive p40 and KIT (CD117); and negative TTF1, neuroendocrine biomarkers and the nuclear protein of the testis (NUT) in immunohistochemistry
Lymphoepithelial carcinoma	Primary thymic carcinoma; Cancer cells are arranged in sheet, nest and strip‐like patterns, with obvious multinuclear, vesicular chromatin and nucleolus; A large number of mixed lymphocytes and plasma cells	Negative Epstein‐Barr Early RNA (EBER) (in situ hybridization) does not affect the diagnosis of typical lymphoepithelial carcinoma morphology; EBER‐negative can support the diagnosis of cases with fewer lymphocytes
NUT carcinoma	*NUTM1* rearrangement or immunohistochemical NUT positive detected in poorly differentiated squamous cell carcinoma or other cancer	
Clear cell carcinoma	Invasive thymic tumor; The cytoplasm of cancer cells is transparent, forming islands and frames; Abundant vitreous stroma in hyaline clear cell carcinoma	Positive cytokeratin, P40 and P63 in immunohistochemistry; *EWSR1* translocation of hyaline clear cell carcinoma
Low grade papillary adenocarcinoma	Low grade, primary thymic adenocarcinoma with tubular papillary growth	Exclusion of thyroid, pleural, lung and extrathoracic papillary tumors by immunohistochemistry
Mucoepidermoid carcinoma	A primary thymic carcinoma characterized by mixing of mucus‐producing cells, intermediate cells and squamous cells in the nested and cystic structures; The diagnosis of high grade mucoepidermoid carcinoma requires at least the presence of focal intracellular mucin	*MAML2* rearrangement can help the diagnosis of complicated cases, especially for high grade cases; Patients with negative *MAML2* rearrangement cannot be excluded
Adenoid cystic carcinoma‐like thymic carcinoma	A thymic carcinoma with morphology similar to adenoid cystic carcinoma, but usually lacks of true gland in cribriform‐basaloid island; Exclude metastatic carcinoma of salivary gland, lung or breast	Negative KIT (CD117) and myoepithelial biomarkers in immunohistochemistry
Intestinal adenocarcinoma	A primary thymic tumor similar to colorectal adenocarcinoma	Expression of at least one intestinal differentiation marker (CD20, CDX2 and MUC2)
Adenocarcinoma Unspecific (NOS)	Exclude mediastinal metastasis of other cancers and defined thymic adenocarcinoma (low grade papillary and intestinal type)	Exclusion of mediastinal metastasis and defined thymic adenocarcinoma by immunohistochemistry
Adenosquamous carcinoma	A thymic carcinoma with both squamous and adenocarcinoma differentiation; Exclude mucoepidermoid carcinoma	
Sarcomatoid carcinoma, Carcinosarcoma	The thymic epithelium, which is partially or completely composed of atypical spindle cells, has at least the characteristics of focal epithelium or heterologous sarcoma‐like components	Immunohistochemistry and molecular detection
Undifferentiated carcinoma	Primary malignant thymic tumor with only epithelium differentiation, and does not conform to other clearly‐defined types; Immunohistochemistry and molecular detection are required	
Thymic carcinoma (NOS)	Exclude the above mentioned thymic cancer types; Currently, this type includes hepatoid carcinoma, rhabdoid carcinoma, undifferentiated large cell carcinoma with Castleman's disease‐like reaction and sebaceous adenocarcinoma	

Abbreviations: TC, thymic carcinoma; TM, thymoma.

Needle biopsy is frequently used for pathological assessment, including fine needle aspiration biopsy (FNA), fiberoptic bronchoscopy or esophagoscopy needle biopsy, ultrasound‐guided mediastinal tumor needle biopsy, CT‐guided percutaneous mediastinal tumor biopsy, etc. All the above techniques are featured by small trauma, simple operation, safe and effective. However, these methods can only obtain a few amount of tissues and are often unable to support accurate pathological diagnosis. These methods also cannot determine the pathological differentiation between TM, lymphoma and thymic hyperplasia. Surgical biopsies, such as mediastinoscopy, thoracoscopy and small incision thoracotomy, are applicable to some complicated advanced patients.

### Molecular diagnosis

Pathological examination is the gold standard for TET classification. With the advances in molecular diagnostic technology and its wide application in clinical practice, researchers have gradually discovered the genomic characteristics of TET. These specific genomic alterations could serve as biomarkers for accurate diagnosis and treatment of TET (Table 2).^5^, ^6^, ^7^, ^8^ *GTF2I* is a unique oncogene of TET with high mutation frequency (about 40%), especially in type A and AB TM.^5^, ^6^ Several researches reported that *GTF2I* mutations in TET are located in the same codon Leu424His (L424H; Leu: ：leucine; His：: histidine). In other solid tumors, *GTF2I* is rarely mutated and its mutations occur in diverse codons.^5^, ^6^ In addition to *GTF2I*, mutations in driving genes such as *TP53*, *KIT*, *ROS1*, *ALK*, *EGFR*, *PI3KCA* and *RAS* family genes may also occur in TET.^5^, ^6^, ^7^, ^9^ Song et al. performed sequencing of 22 cancer‐related hotspot genes in 55 Chinese TET patients, and discovered two *PI3KCA* mutations and one *EGFR* mutation.^10^ Recently, a study targeting frequently mutated genes of 15 solid tumors revealed the mutation profile of 53 TET patients. This study demonstrated that 29.4% of TC carried at least one pathogenic mutation in *TP53*, *ERBB2*, *KIT* and *KRAS* genes.^7^ *KIT* L576P mutated patients show low to moderate sensitivity to imatinib, sunitinib, dasatinib and nilotinib. Molecular testing such as multigene next‐generation sequencing can detect actionable mutations in TET and guide targeted therapies for patients. This provides more treatment options in addition to conventional treatments, especially for patients with inoperable advanced diseases. Unfortunately, limited number of targeted therapies can be used according to the existing molecular profiles of TETs.^6^, ^7^ Hopefully, the analysis of mutation characteristics of TETs and the stratification of patients based on molecular and histopathological characteristics may promote the research and development of drugs and lead to the precision medicine of TETs.^11^

**TABLE 2:** Molecular alterations in thymic epithelial tumor (TET).

WHO classification	Structural variation	Gene mutation
Type A	Chromosome deletion: 6q25.2–25.3, 2, 4, 6q, 13, 6p21 Chromosome translocation: t (15;22) (p11; q11)	*GTF2I*, *TP53*, *HRAS*, *EGFR*, *STK11*, *SMARCB1*, *TET2*, *PDGHRA*, *RUNX1*, etc.
Type AB	Chromosome deletion: 2.4, 5q21‐22, 6p21, 6q25.2–25.3, 7p15.3, 8p, 13q14.3, 16q, 18	
Type B1	Chromosome deletion: 1p, 1q, 3q, 4, 5, 6q, 8, 13, 18 Chromosome duplication: 9q	
Type B2	Chromosome deletion: 6q25.2–25.3和3p Chromosome duplication: 1q	
Type B3	Chromosome deletion: 3p, 6, 6q25.2–25.3, 9p, 11q42.qter, 13q, 16q, 17p, 9q21.3 (*CDKN2A*/*CDKN2B*) Chromosome duplication: 1q, 4, 5, 7, 8, 9q, 17q, X, 18q21.33 (*BCL2*)	
TC (type C)	Chromosome deletion: 3p, 6, 6q25.2–25.3, 9p, 13q, 14, 16q, 17p, 9q21.3 (*CDKN2A*/ *CDKN2B*) Chromosome duplication: 1q, 4, 5, 7, 8, 9q, 12, 15, 17q, 18, 20, 18q21.33 (*BCL2*)	*GTF2I*, *TP53*, *KIT*, *KRAS*, *NRAS*, *ROS1*, *DDR2*, *PDGFRA*, *IGF1R*, *ERBB2*, *ROS1*, *ALK*, *ATM, CDKN2A*, *FGFR3*, *HRAS*, *PTEN*, etc.

Abbreviations: TC, thymic carcinoma.

## SURGICAL TREATMENT

Consensus 5: TET patients with Masaoka‐Koga stage I–IIIA are suitable for surgical treatment. Therefore, for patients with operable early TET, surgical treatment is recommended (strongly recommended).

At present, the treatment decision of TET is mainly based on the surgical resectability and whether complete resection can be done. Before the establishment of a well‐developed treatment strategy based on TNM staging, Masaoka‐Koga stage is generally used to guide clinical treatment. The TNM stage and Masaoka‐Koga stage are partly correlated, and treatment strategies can be decided by considering both of them. TNM staging with American Joint Committee on Cancer (AJCC) version 8 is recommended. (Tables S1–S3).

For Masaoka‐Koga stage I–IIIA TET, the first choice of treatment is surgery. Complete removal of the thymus, including thymic tumor, residual thymus and surrounding fat tissue is recommended.^12^ For operable patients with advanced TET, adjacent tissues, pericardium, phrenic nerve, pleura, lung and major vascular structures should be removed.

A surgical approach through the median sternal incision is recommended. Capsular invasion, thymic peripheral infiltration, pleural and peripheral structure involvement can be evaluated by the median sternal incision. This surgical approach can also maintain the organ tissue structure of the mediastinum.^13^ During the operation, the regional lymph nodes are removed, and the anterior mediastinal lymph nodes and anterior cervical lymph nodes routinely cleaned.

At present, minimally invasive surgery is mainly used for the surgical treatment of early tumors. Minimally invasive surgery is an effective alternative to open surgery, and it has many advantages over open surgery, including reduce in blood loss and complications, as well as shorter hospital stay and lower rate of positive margin.^14^ However, there is no significant difference between the survival of patients receiving minimally invasive surgery and open surgery. In a retrospective analysis of 280 patients with TETs who received surgical treatment, the 5‐year relapse‐free survival (RFS) rate in the thoracoscopic surgery group was 93.8%, and the 5‐year OS rate was 97.9%. There were also no significant differences between the thoracoscopic surgery group and the sternotomy group in RFS and OS (*p* = 0.91 and *p* = 0.74).^15^ In another study of early TM treated by surgery, there was no significant difference in OS, RFS and time of tumor recurrence between the thoracoscopic surgery and the sternotomy groups.^16^

## RADIOTHERAPY

Consensus 6: For unresectable TET (including disease progression after preoperative neoadjuvant therapy) or incomplete surgical resection of TET, radical concurrent chemoradiotherapy (CRT) should be performed. For locally advanced TET (about 40% of patients still recurred after R0 resection), adjuvant postoperative radiotherapy (PORT) is recommended (strongly recommended).

Consensus 7: For unresectable advanced TET with poor chemosensitivity, induction radiotherapy and post‐recurrence radiotherapy can be considered (strongly recommended).

### Indication for radiotherapy

Despite the lack of high‐level evidence, unresectable TET patients are generally treated with radical concurrent CRT or radical radiotherapy. At present, most studies are retrospective, apart from two prospective phase II studies. Loehrer's landmark prospective study recruited 23 TET patients who received induction chemotherapy with cyclophosphamide, doxorubicin and cisplatin, followed by radiotherapy with a total dose of 54 Gy.^17^ The response rate of this regimen was 70%, and the 5‐year OS was 53%. The largest prospective phase II study conducted by Fan et al. included 56 patients and evaluated the efficacy of concurrent CRT with etoposide/cisplatin (EP) as the first‐line treatment of advanced TETs (85.7% stage IV). The results showed that 54 Gy of intensity‐modulated radiotherapy (IMRT) combined with EP regimen was safe and effective for unresectable TETs. The objective response rate (ORR) was 85.7%, and the 5‐year PFS and OS rates were 29.5 and 56.2%, respectively.^18^

PORT is also considered as an important part of the treatment of TET. However, the best strategy for this regimen remains controversy. At present, adjuvant radiotherapy is not recommended for stage I patients after R0 resection. It is still controversial whether stage II–III patients with R0 resection should receive adjuvant radiotherapy, especially for stage II patients after R0 resection. Several clinical studies have shown that PORT can improve local control and survival for stage II and R0 resected patients who have high risk factors such as proximal incisional margin or invasive histology (type B2, type B3, TC) or extensive capsular invasion (stage IIB). In a study involving 1320 Masaoka stage II TM patients who received surgical treatment, 170 patients achieved complete resection. Among them, no statistically significant difference in rate of recurrence was observed between those patients receiving surgical treatment (26%, 8/31) and PORT (23%, 18/78).^19^ Based on a retrospective analysis of 4056 cases of TET included in the NCDB database from 2004 to 2012, it was found that patients with Masaoka stage I and stage IIA had no significant benefit from PORT compared with surgery alone, while patients with stage IIB (*p* = 0.035) and stage III (*p* = 0.020) showed a longer survival when receiving adjuvant PORT.^20^ Another study involving 1263 patients with R0‐resected, stage II to III TM also showed that PORT significantly prolonged patient survival (*p* = 0.002).^21^ For TC, a study showed that Masaoka stage II–III patients can benefit from PORT.^22^ This study did not exclude patients with incomplete resection, but the majority of them had complete resection. Interestingly, this study also found that the efficacy of PORT was limited in patients with stage II–III TM. In addition, a meta‐analysis showed that PORT could improve the OS (*p* < 0.001) and progression‐free survival (PFS) (*p* < 0.001) in patients with stage I–IV TC.^23^ In summary, radiotherapy after complete resection is not recommended for stage I TM, and is inconclusive for stage II TM. Many studies have supported the use of PORT in stage III TM. Adjuvant PORT may be considered for TM after complete resection.

A study in 2021 combined the data from five observational studies published since 2013 for meta‐analysis. The study evaluated 4746 patients with completely resected, Masaoka‐Koga stage II TM (equivalent to TNM stage I–III) and showed that PORT significantly improved the OS of this population (hazard ratio = 0.68, 95% confidence interval: 0.57–0.83, *p* < 0.001). Based on the results of these currently available observational studies, PORT is suitable for patients with completely resected, Masaoka‐Koga stage II TM.^24^ The propensity score matching analysis with data from SEER database showed that PORT improved the OS and cancer‐specific survival of patients with Masaoka‐Koga stage IIB–IV TM, but had no significant survival benefit for patients with stage I–IIA TM. This study also proved the prognostic benefit of PORT in stage IIB TC for the first time.^25^

A prospective study conducted in 22 patients with locally advanced TET (stage III/IV) evaluated the efficacy of neoadjuvant CRT. Most (77%) patients can achieve R0 resection after neoadjuvant CRT. There is a higher OS benefit for downstaged patients.^26^ Therefore, induction CRT may be a feasible option to achieve complete resection in patients with locally advanced and highly invasive tumor, further improving the rate of survival. If the tumor can be removed after neoadjuvant chemotherapy, surgical treatment should be performed for stage IIIA patients. Additional PORT should be performed for stage IVB patients. The recommended radiotherapy dose for R0, R1, and R2 resected patients are 45–50 Gy, 54 Gy, and 60–70 Gy, respectively. For R2 resected, stage IVB patients, platinum/etoposide CRT should be performed. If the tumor cannot be removed after neoadjuvant chemotherapy, neoadjuvant or radical radiotherapy (60–70 Gy) can be considered for stage IVB patients, with or without platinum/etoposide concurrent chemotherapy.^27^

### Analog positioning

It is recommended to plan for radiotherapy based on CT. CT scan should be performed according to the tumor location. For the most common upper mediastinal tumors, head and neck shoulder film fixation can be used, with both hands placed on the body side. For lesions with a wide head angle, patients should lift their arms and place it on the forehead, and body membrane can be used for fixation. It is encouraged to simulate the target area movement. The target area movement should be handled according to the radiotherapy principles in the guidelines of the NCCN (non‐small cell lung cancer) of the United States. If four‐dimensional (4D) CT is available, it is recommended to obtain and determine the tumor movement and respiration (internal edge) in order to improve the volume limit. 18F‐FDG PET/CT may help to improve the accuracy of the target area in certain circumstances.

### Radiation techniques

At least three‐dimensional conformal radiotherapy techniques should be used to reduce damage to surrounding normal tissues (such as heart, lung, esophagus and spinal cord). Intensity modulated radiotherapy (IMRT), the current standard method for radiotherapy, can further optimize the distribution of radiation dose and reduce the damage to normal tissues. A retrospective study comparing IMRT/3D‐CRT with traditional radiotherapy found that patients treated with IMRT/3D‐CRT had better survival outcomes due to smaller radiation field, higher accuracy and lower dose to healthy organs.^28^ The combination of IMRT and 4D‐CT simulation can further reduce the radiation‐induced toxicity, leading to better local control and OS.^29^, ^30^ Research showed that, compared with IMRT, proton beam radiotherapy (PBT) can improve the distribution of radiotherapy dose and better protect normal organs. In addition, PBT showed good results in local control and safety profiles.^31^, ^32^, ^33^, ^34^, ^35^ In a prospective study of 27 patients receiving proton therapy, the local control rate was 100% and the 3‐year OS rate was 94% (median follow‐up of 2 years). The toxicity was mild to moderate, with no grade 3 or higher adverse event observed.^36^ Based on these evidences, it is suggested to consider PBT if conditions permit.

### Target area and radiation dose

The target area of radical radiotherapy should include all the tumors visible to the naked eye. The target area of adjuvant PORT should include the whole tumor bed according to the preoperative imaging. Radiologists need to communicate with surgeons about intraoperative findings to help determine the target area. The retention of metal clips during surgery can assist in determining the surgical resection boundary and/or the location of nonresectable residual lesions, which plays an important role in the planning of target area for PORT. Also, radiologists should communicate with pathologists about the histological morphology of the lesion, the degree of invasion (such as the degree of extracapsular invasion) and the surgical margin status. Target areas with incompletely resected tumors include resection of the tumor bed, the residual thymus and all the potential area of residual lesions. Lymph node metastasis is rare in TET, and thus extensive lymph node irradiation is not recommended. In contrast, pleural metastasis occurs frequently in TET. For TC patients with pleural metastasis, hemithoracic radiotherapy can be considered. Gross tumor volume (GTV) to clinical target volume (CTV): 0.5 to 1.0 cm. CTV to planning target volume (PTV) (conventional CT): 1.0 to 1.5 cm. Internal target volume (ITV) to PTV (4D‐CT, without image‐guided radiotherapy technique [IGRT]): 0.5 to 1.0 cm. ITV to PTV (4D‐CT and IGRT): 0.5 cm.

The NCCN guideline for TET has recommendations on the radiation dose and fractionation scheme. It is suggested that patients with unresectable TET are treated with radical radiotherapy at a dose of 60–70 Gy. For radical radiotherapy after incomplete resection, the recommended doses for pathological negative margin, microscopic positive margin and macroscopic positive margin are 45–50, 54 and 60–70 Gy, respectively. For adjuvant PORT in locally advanced tumors, the recommended dose is 40–50 Gy, with a fractionation scheme of 1.8–2.0 Gy each time, lasting for 4–6 weeks. Palliative radiotherapy can be performed for advanced tumors: palliative doses (for example, 8 Gy in a single fraction, 20 Gy in five fractions, 30 Gy in 10 fractions) up to definitive doses (60–70 Gy, conventional fractionation) can be applied to achieve long‐term local control. For metastatic lesions with limited volume, it is recommended to use highly conformal radiotherapy techniques. Stereotactic radiotherapy techniques can be used if conditions permit.

### Normal tissue dose constraints

The radiation dose constraints in TM are the same as that in lung cancer. Nevertheless, it is recommended that the normal tissue dose constraints are reduced as much as possible because the prognosis of TM is generally better than that of lung cancer. Especially when anthracycline drugs are used for chemotherapy, the cardiac dose should be more limited **(**Table 3 **)**.

**TABLE 3:** Radiation dose constraints in thymoma (TM).

	Radiotherapy alone	CRT	Preoperative CRT
Spinal cord	Dmax <45 Gy	Dmax <45 Gy	Dmax <45 Gy
Lung	MLD ≤20 Gy	mean lung dose (MLD) ≤20 Gy	MLD ≤20 Gy
	V20 ≤ 40%	V20 ≤ 35%	V20 ≤ 30%
		V10 ≤ 45%	V10 ≤ 40%
		V5 ≤ 65%	V5 ≤ 55%
		V30 ≤ 45%	V30 ≤ 45%
Heart	V30 ≤ 40%	V30 ≤ 40%	V30 ≤ 40%
	Mean dose <26	Mean dose <26 Gy	Mean dose <26 Gy
Esophagus	Gy	Dmax ≤80 Gy	Dmax ≤80 Gy
	Dmax ≤80 Gy	V70 < 20%	V70 < 20%
	V70 < 20%	V50 < 40%	V50 < 40%
	V50 < 50%	Mean dose <34 Gy	Mean dose <34 Gy
	Mean dose <34 Gy		

Abbreviation: CRT, concurrent chemoradiotherapy.

### Radiotherapy complications

Early acute reactions: skin reaction, fatigue, dysphagia, pharyngalgia, cough, dyspnea, L'hermite syndrome (radiation myelitis), acute radiation pneumonia.

Late reactions: pericarditis, restrictive cardiomyopathy, myocardial infarction, chronic heart failure, radiation cardiomyopathy, radiation pneumonia and pulmonary fibrosis.

## MEDICAL TREATMENT

Consensus 8: Routine adjuvant chemotherapy is not recommended for Masaoka‐Koga stage I–III TET after R0 resection. For R1 or R2 resected TET treating with PORT, the use of combination chemotherapy should be considered according to the tumor stage, tumor invasiveness and the patient's physical condition (strongly recommended).

Consensus 9: The symptoms and signs of MG should be evaluated and well‐controlled for all the TET patients before surgery. Myasthenia crisis is a common post‐surgery complication of TET accompanied with MG. Individualized regimen modification, early identification and timely and effective treatment are the key to prevent the occurrence of myasthenia crisis (strongly recommended).

Consensus 10: For TET initially evaluated to be unresectable, induction chemotherapy is recommended, and subsequent surgery or radiotherapy should be considered according to the prognosis. For TET with evidence of extrathoracic metastasis, platinum‐based systemic chemotherapy is recommended. Among them, first‐line treatment with cyclophosphamide + doxorubicin + cisplatin is the first choice for TM, and first‐line treatment with carboplatin + paclitaxel is the first choice for TC. Targeted drugs show certain efficacy in TET progressed after first‐line chemotherapy and can be used in patients with disease progression or intolerable to first‐line chemotherapy. Second‐line pembrolizumab shows good antitumor activity, but the incidence of immune‐related side effects is high. It should be carefully considered in TC and is not recommended in TM. Immune checkpoint inhibitors combined with chemotherapy may improve the antitumor effect (strongly recommended).

The medical treatment of TET consists of the treatment of the tumor itself and the management of its complications. The treatment part should be decided according to the tumor stage, pathological type and post‐surgery residual tumor status. MG is the main complication of TET. About 30%–50% of TM patients have TET accompanied by MG.^37^ This is the direct cause of death of some patients. However, MG is rare in patients with TC.

### Management of MG

Poor control of MG before surgery is an important reason for postoperative myasthenia crisis. Prior to surgery, MG should be medically controlled with careful evaluation and consultation with neurologists. The evaluation should mainly include medical history, symptoms, physical conditions and MG‐related pathogenic antibody tests.

There are two main types of routine therapeutic drugs used before surgery: cholinesterase inhibitors and immunosuppressive drugs. Intravenous injection of gamma globulin and plasma exchange are mainly used in patients with acute progression or severe symptoms. The dosage of cholinesterase inhibitor should be individualized, and the optimal dosage is the one that can maintain normal living ability. It is recommended to continue the original dosage before surgery. When combined with glucocorticoids or other immunosuppressive drugs, it is suggested to adjust the dosage of glucocorticoids to the lowest effective dose before surgery, or stop using the drug completely; Other drugs such as azathioprine, cyclophosphamide, mycophenolate mofetil and tacrolimus should be stopped for more than 2 weeks before surgery.^38^

The adjustment of postoperative dosage of cholinesterase inhibitor generally begins from half the presurgery dosage, and individualized adjustment is made according to the patient's symptoms. Immunosuppressants such as glucocorticoids can be flexibly used in combination, and the dosage can be gradually reduced or stopped when the expected effect has been achieved. Dysphagia, bulbar muscle weakness, increased oral secretion, choking and difficulty in expectoration are common early symptoms of crisis. These symptoms should be closely examined and actively treated. For patients with myasthenia crisis, large‐dose and short‐course hormone pulse therapy can be considered. Intravenous injection of immunoglobulin or plasma exchange can be used if necessary.^38^

### Systemic chemotherapy

The prognosis of TET, especially TC, is closely related to the tumor stage and the resection margin status. TETs are generally insensitive to chemotherapy, and the benefit of adjuvant chemotherapy is not obvious.^39^, ^40^ Therefore, it is not recommended to apply adjuvant chemotherapy to patients with Masaoka‐Koga stage I–III after R0 resection. For stage III patients without preoperative neoadjuvant chemotherapy, adjuvant chemotherapy may be considered.

For R1 resected TM, radiotherapy alone can be used. For R1 resected TC or R2 resected TM, the use of combination chemotherapy should be considered according to the tumor stage and the patient's physical condition. For R2 resected TC, radical CRT is recommended.^41^, ^42^

Induction chemotherapy is an important treatment option that could improve the complete resection rate of potentially resectable TET. Between 2–4 cycles of induction chemotherapy may reduce the tumor volume and make the tumor resectable. Cisplatin‐based combination regimen is recommended as the induction chemotherapy for TM, of which the CAP regimen (cyclophosphamide + doxorubicin + cisplatin) and EP regimen (etoposide + cisplatin) are preferred. Etoposide and platinum‐based concurrent CRT can be considered for TC patients.

Recently, a large cohort study reported that the 5‐year OS of patients with surgical resection after induction chemotherapy was comparable to that of patients receiving direct surgical resection (77.4% vs. 76.7%; *p* = 0.596).^43^ After induction chemotherapy, the whole‐body imaging assessment should be performed again. If the lesions can be completely removed, it is recommended to remove the primary lesion and the oligo‐metastatic lesions by surgery; If the lesion is still unresectable or disease progression occurs, radical radiotherapy or concurrent CRT is recommended.^43^, ^44^, ^45^, ^46^, ^47^

Because of the rarity and the lack of randomized controlled study of TET, the standard chemotherapy regimen remains unclear, and its benefit in prolonging survival requires further validation. The main purpose of chemotherapy is to reduce the tumor load and relieve tumor‐related symptoms.

The first‐line CAP regimen (cyclophosphamide + doxorubicin + cisplatin) is the first choice for treating advanced TM. Studies suggested that this regimen has better treatment outcomes than other regimens,^48^, ^49^, ^50^, ^51^ with a response rate of 44%.^52^ For patients who cannot tolerate the CAP regimen, nonanthracycline combination regimen can be used, such as cisplatin + etoposide, or carboplatin + paclitaxel. Other first‐line treatment options include CAP plus prednisone, cisplatin + doxorubicin + vincristine + cyclophosphamide (ADOC) and etoposide + cyclophosphamide + cisplatin.

The first‐line choice for treating advanced TC is carboplatin + paclitaxel, with an overall response rate of 22%–36%.^53^, ^54^, ^55^, ^56^ Although CAP and cisplatin + doxorubicin + vincristine + cyclophosphamide ADOC are also effective, these regimens show higher toxicity.

For patients with TET who cannot tolerate first‐line regimens, second‐line regimens with low toxicity can also be used. However, there are limited evidences to support the recommendation on the preferred regimen. The commonly used regimens include fluorouracil + tetrahydrofolate, gemcitabine ± capecitabine, pemetrexed and paclitaxel (Tables S4 and S5).

### Targeted therapy

The number of actionable alterations in TET is limited. Currently, there is a lack of evidence‐based targeted therapy. Less than 10% of TC harbors *c‐kit* mutations, while almost no *c‐kit* mutations are found in TM. Sunitinib and lovatinib are oral tyrosine kinase inhibitors with multiple targets such as *KIT*/*PDGFR*/*VEGFR*. They are only recommended for second‐line treatment of TC, regardless of the presence of *c‐kit* mutation. In a phase II clinical trial, sunitinib was administered orally, 50 mg daily for the first 4 weeks and stopped for the next 2 weeks, every 6 weeks as a cycle. For the 23 cases of TC treated by sunitinib, six cases had partial response (26%), 15 cases had stable disease (65%) and two cases had disease progression (9%). For the 16 cases of TM treated by sunitinib, one case had partial response (6%), 12 cases had stable disease (75%) and three cases had disease progression (19%).^57^ Sunitinib shows a better ORR in TC. A recent study adopting a continuous oral administration also confirmed that the ORR of sunitinib was better in TC (41.7% vs. 14.3%), but the disease control rate was better in TM (100% vs. 75%), indicating that the response rate might be related to the tumor invasiveness.^58^ In a phase II clinical study recruiting advanced or metastatic TC, 42 patients were treated with lovatinib, 16 patients had partial response, 24 patients had stable disease, the ORR was 38%, and the disease control rate was 95%.^59^

Everolimus, a mammalian target of rapamycin (mTOR) inhibitor, has also achieved good efficacy in TET. In a study recruiting patients with advanced TM and TC who progressed after platinum‐based chemotherapy, the disease control rate of TC was 88%, and the median PFS and OS were 10.1 months and 25.7 months, respectively.^60^

Apatinib is an angiogenesis inhibitor targeting VEGFR‐2. A phase II clinical study recruiting patients with recurrent or metastatic TET found that the ORR and disease control rate of apatinib reached 40% and 84%, respectively, and the median PFS and OS were 9 months and 24 months, respectively, showing a good antitumor effect.^61^

Cixutumumab is a monoclonal antibody targeting insulin‐like growth factor 1 receptor. It has a good efficacy in TM progressed after chemotherapy. In a previous study, the response rate was 14% (5/37) and the disease control rate was 89.2% (33/37). However, none of the patients with pretreated TC achieved disease remission.^62^

### Immunotherapy

PD‐L1 is highly expressed in TET. The reported positive rate ranges from 23% to 92%. In a phase II clinical study (*n* = 40) evaluating the efficacy of the PD‐1 inhibitor pembrolizumab in recurrent TC, one case (2.5%) had complete response, eight cases (20.0%) had partial response, 21 cases (22.5%) had stable disease, and the ORR reached 22.5%.^63^ Similarly, a study involving 26 patients with TC who received immunotherapy showed an ORR of 19.2%.^64^ In addition, a phase II clinical study involving 15 cases of unresectable or recurrent TC treated with nivolumab monotherapy reported that eight patients had stable disease and none of the patients achieved remission. The latest retrospective study suggested that immune checkpoint inhibitor combined with chemotherapy can significantly improve the ORR (44.4%) and extend the PFS. The incidence of grade 3–4 adverse reactions was 15.6%.^65^ Prospective, randomized controlled trials are required to further validate these results.

The incidence of grade 3–4 immune‐related adverse reactions in TET patients treated with immune checkpoint inhibitors was 15%,^63^ and even reached about 70% (5/7) in TM.^64^ The incidence of severe myocarditis was 5% to 9%.^63^, ^64^ In a retrospective study of 11 patients with TM who received immunotherapy, the incidence of immune‐mediated myocarditis reached 36.4%.^66^ Therefore, it is necessary to closely monitor the immune‐related adverse reactions during the course of immunotherapy in TET, especially for patients with autoimmune syndrome. Before receiving immunotherapy, it is necessary to balance the benefits and risks of the treatment.

## PALLIATIVE AND SUPPORTIVE TREATMENTS

Consensus 11: Pain management and relieving symptoms of dyspnea and hypoxia are the key of palliative and supportive treatments for patients with TET. Medical treatment is used for cancer pain management; For patients who cannot receive radical surgery, tumor reduction surgery with parenchyma preservation can be considered (strongly recommended).

Medical treatment is the first choice for cancer pain management of TET patients. For moderate pain, weak opioids such as codeine or tramadol can be added; For severe pain, strong opioids can be added;^67^ Cancer‐induced bone pain and neuropathic pain are difficult to control due to the local effect on the neurovascular bundle. In addition to opioids, auxiliary drugs targeting specific neuropathic pain mechanisms can also be used. Ticyclic antidepressants and antiepileptic drugs are the most commonly used drugs.^67^, ^68^

Palliative surgery can be used to treat TET patients. The indication for palliative surgical treatment includes patients who are not suitable for macroscopic resection due to tumor stage or disease status.

## FOLLOW‐UP

Consensus 12: Chest CT as the baseline examination should be performed 3–4 months after surgical resection of TET. TM and TC patients should be followed up for 5 years and 10 years, respectively (strongly recommended).

Several retrospective studies reported that WHO histologic subtypes, Masaoka stage, TNM stage and surgical margin are independent prognostic factors of disease‐free survival (DFS) and OS in TET patients.^69^ At present, patients are divided into low‐ and high‐risk groups, mainly based on histological subtypes, for differential clinical management with corresponding follow‐up strategies. According to a previous statistical report, the 10‐year OS of patients was 89.5%, of which 5.8% had distant and/or local‐regional recurrence. The recurrence rate of the low‐risk group was significantly lower than that of the high‐risk group. In the high‐risk group, more than half of the patients relapsed within 3 years after surgery, and almost all the patients relapsed within 6 years after surgery. In the low‐risk group, the recurrence events were evenly distributed within 10 years after surgery.^70^ Therefore, for high‐risk patients, it is recommended that re‐examination is performed every 6 months for the first 3 years after surgery, and every year thereafter, until recurrence. For low‐risk patients, it is recommended that re‐examination is performed once a year, until 10 years after surgery. It should be noted that low‐risk patients are still at risk of developing secondary malignant tumors.

The OS and RFS rates are lower in patients with higher Masaoka stage.^71^, ^72^ The widely used TNM staging system for TET is also related to survival of patients: the 5‐year OS of stage I–IV, completely resected patients are 90, 90, 60 and 25%, respectively.^73^, ^74^, ^75^ Therefore, corresponding re‐examination strategies, mainly based on CT scan, can also be decided according to clinical stages. First, it is recommended to perform chest CT scan 3–4 months after surgery, serving as the baseline examination. For patients with completely resected, stage I/II TM, CT scan should be performed once a year for the first 5 years, and every 2 years thereafter; For patients with stage III/IV TM, TC or R1‐2 resection, CT scan should be performed every 6 months for the first 2 years, and once a year thereafter. Follow‐up can last for 10 years.^27^

For patients receiving postoperative adjuvant treatment or with advanced TET, the frequency and method of re‐examination can be adjusted according to the actual situation. Clinicians should also pay attention to new developed autoimmune diseases that may occur late. For patients with MG or even with positive antiacetylcholine receptor antibody, they should be informed and educated about the risk of myasthenia crisis under specific circumstances such as stress and medication. Additional stress management is required in some cases.

## AUTHOR CONTRIBUTIONS

Anwen Liu, Wenfeng Fang and Wenzhao Zhong participated in the design of the expert consensus. Chunwei Xu, Yongchang Zhang, Wenxian Wang and Qian Wang conceived of the expert consensus, and participated in its design and other authors coordination and helped to draft the expert consensus. All authors read and approved the final manuscript.

## CONFLICT OF INTEREST STATEMENT

There are no conflicts of interest to disclose.

## Supporting information


**APPENDIX S1:** Supplementary Information.
