# Supplementary material for: Chinese expert consensus on the diagnosis and treatment of thymic epithelial tumors
Source: Thorac Cancer. 2023 Mar 16;14(12):1102–17. doi: 10.1111/1759-7714.14847 (PMC10125784; doi:10.1111/1759-7714.14847)
Supplement: Supplementary file 1 — APPENDIX S1: Supplementary Information. [file TCA-14-1102-s001.docx]

**Appendix - Staging**

Masaoka staging system was first proposed in 1981. It is currently the most widely used staging system. The staging system classifies tumors according to the capsular status of surgical specimen and the scope of tumor invasion, and is predictive for the clinical outcome of TM and TC. The International Association for the Study of Lung Cancer (IASLC) and the International Thymic Malignancy Interest Group (ITMIG) recommend to simultaneously apply Masaoka staging system and TNM staging system for TET staging (Supplementary Tables 1, 2 and 3)^1, 2^.

**Supplementary Table 1. Masaoka-Koga staging system for TET**

| Masaoka stage | Diagnostic criteria |
| --- | --- |
| Stage I | Macroscopically and microscopically completely encapsulated tumor |
| Stage II | 1. Microscopic transcapsular invasion |
|  | 1. Macroscopic invasion into surrounding fatty tissues,   or grossly adherent to but not through mediastinal pleura or pericardium |
| Stage III | Macroscopic invasion into neighboring organs (i.e.  pericardium, great vessels or lungs) |
|  | 1. Without great vessel invasion |
|  | (B) With great vessel invasion |
| Stage IV | 1. Pleural or pericardial metastasis |
|  | 1. Lymphogenous or hematogenous metastasis |

**Supplementary Table 2. TNM staging system**

| Primary tumor (T) | |
| --- | --- |
| TX | Unable to assess primary tumor |
| T0 | No evidence of primary tumor |
| T1 | Encapsulated tumor or extending into mediastinal fat; may involve mediastinal pleura |
|  | T1a：tumor without mediastinal pleura involvement |
|  | T1b：tumor with mediastinal pleura involvement |
| T2 | Tumor with direct invasion into pericardium (partial or full thickness) |
| T3 | Tumor with direct invasion into any of the following structures: lung, brachiocephalic vein, superior vena cava, phrenic nerve, chest wall, extrapericardial pulmonary artery or vessels |
| T4 | Tumor with invasion into any of the following structures: aortic arch vessels (ascending aorta, aortic arch or descending aorta), intrapericardial pulmonary artery, myocardium, trachea, esophagus |
| Regional lymph nodes (N) | |
| NX | Unable to assess regional lymph nodes |
| N0 | No regional lymph node metastasis |
| N1 | Anterior (perithymic) lymph node metastasis |
| N2 | Deep intrathoracic or cervical lymph node metastasis |
| Distant metastasis (M) | |
| M0 | No pleural, pericardial or distant metastasis |
| M1 | Pleural, pericardial or distant metastasis |
|  | M1a：Separate pleural or pericardial nodule(s) |
|  | M1b：Pulmonary intraparenchymal nodules or distant organ metastasis |

**Supplementary Table 3. AJCC** **prognostic groups**

| Stage I | T1a, b | N0 | M0 |
| --- | --- | --- | --- |
| Stage II | T2 | N0 | M0 |
| Stage IIIA | T3 | N0 | M0 |
| Stage IIIB | T4 | N0 | M0 |
| Stage IVA | Any T | N1 | M0 |
|  | Any T | N0-N1 | M1a |
| Stage IVB | Any T | N2 | M0-M1a |
|  | Any T | Any N | M1b |

**Appendix - Medical treatment regimens**

**Supplementary Tables 4. Recommended medical treatment**

| Disease type | Treatment line | Preferred regimen | Other regimens |
| --- | --- | --- | --- |
| TM | First-line | Cyclophosphamide + doxorubicin + cisplatin | Cyclophosphamide + doxorubicin + cisplatin + prednisone, cisplatin + doxorubicin + vincristine + cyclophosphamide, etoposide + cisplatin, etoposide + cyclophosphamide + cisplatin, paclitaxel + carboplatin |
|  | Second-line or late-line | Apatinib | Etoposide, everolimus, fluorouracil+calcium folinate, gemcitabine ± capecitabine, cyclophosphamide, octreotide (including long-acting octreotide) ± prednisone, pemetrexed, paclitaxel |
| TC | First-line | Paclitaxel + carboplatin | Cyclophosphamide + doxorubicin + cisplatin, cyclophosphamide + doxorubicin + cisplatin + prednisone, cisplatin + doxorubicin + vincristine + cyclophosphamide, etoposide + cisplatin, etoposide + cyclophosphamide + cisplatin |
|  | Second-line or late-line | Apatinib | Everolimus, fluorouracil + calcium folinate, gemcitabine + capecitabine, levatinib, octreotide (including long-acting octreotide) ± prednisone, paclitaxel, pebrolizumab, pemetrexed, sunitinib, etoposide^a^, ifosfamide^a^ |

Note: ^a^ Optional under specific circumstances

**Supplementary Table 5. First-line combination chemotherapy commonly used in TET^a^**

| Disease type | Recommended regimen | Administration method | Treatment cycle |
| --- | --- | --- | --- |
| TM | Cyclophosphamide + doxorubicin + cisplatin ^b^ | Cyclophosphamide 500 mg/m², intravenous drip, day 1  Doxorubicin 50mg/m², intravenous drip, day 1  Cisplatin 50mg/m², intravenous drip, day 1 | Once every 3 weeks |
| TC | Carboplatin + paclitaxel ^c^ | Carboplatin, area under the curve (AUC) 5, intravenous drip, day 1  Paclitaxel 175mg/m², intravenous drip, day 1 | Once every 3 weeks |
| TM and TC | Cyclophosphamide + doxorubicin + cisplatin + prednisone ^d^ | Cyclophosphamide 500mg/m², intravenous drip, day 1  Doxorubicin 20mg/m², intravenous drip, day 1-3  Cisplatin 30mg/m², intravenous drip, day 1-3  Prednisone 100mg/d, day 1-5 | Once every 3 weeks |
|  | Cisplatin + doxorubicin + vincristine + cyclophosphamide ^d^ | Cisplatin 50mg/m², intravenous drip, day 1  Doxorubicin 40mg/m², intravenous drip, day 1  Vincristine 0.6mg/m², intravenous drip, day 3  Cyclophosphamide 700mg/m², intravenous drip, day 4 | Once every 3 weeks |
|  | Etoposide + cisplatin ^d^ | Etoposide 120mg/m², intravenous drip, day 1-3 ^e^  Cisplatin 60mg/m², intravenous drip, day 1 | Once every 3 weeks |
|  | Etoposide + cyclophosphamide + cisplatin ^d^ | Etoposide 75mg/m², intravenous drip, day 1-4  Isocyclophosphamide 1.2g/m², intravenous drip, day 1-4  Cisplatin 20mg/m², intravenous drip, day 1-4 | Once every 3 weeks |

Note: ^a^ If the patient cannot tolerate first-line combination treatment, second-line treatment can be considered; ^b^ The first choice for TM can be used as other options for TC; ^c^ The preferred regimen of TC can be used as alternative options for TM; ^d^ Alternative options for TC and TM; ^e^ The overall dose commonly used in China: cisplatin 75mg/m², intravenous drip, administered in day 1-2; etoposide 100mg/m², intravenous drip, day 1-3, once every 3 weeks.

1. Detterbeck, F. C.; Nicholson, A. G.; Kondo, K.; Van Schil, P.; Moran, C., The Masaoka-Koga stage classification for thymic malignancies: clarification and definition of terms. *Journal of thoracic oncology : official publication of the International Association for the Study of Lung Cancer* **2011,** *6* (7 Suppl 3), S1710-6.

2. Detterbeck, F. C.; Stratton, K.; Giroux, D.; Asamura, H.; Crowley, J.; Falkson, C.; Filosso, P. L.; Frazier, A. A.; Giaccone, G.; Huang, J.; Kim, J.; Kondo, K.; Lucchi, M.; Marino, M.; Marom, E. M.; Nicholson, A. G.; Okumura, M.; Ruffini, E.; Van Schil, P., The IASLC/ITMIG Thymic Epithelial Tumors Staging Project: proposal for an evidence-based stage classification system for the forthcoming (8th) edition of the TNM classification of malignant tumors. *Journal of thoracic oncology : official publication of the International Association for the Study of Lung Cancer* **2014,** *9* (9 Suppl 2), S65-72.
